# Evaluation of the Biodegradability Potential of Antibacterial Poly(lactic acid)/Glycero-(9,10-trioxolane)-trialeate Films in Soil

**DOI:** 10.3390/polym18020216

**Published:** 2026-01-13

**Authors:** Olga V. Alexeeva, Yulia V. Tertyshnaya, Sergey S. Kozlov, Vyacheslav V. Podmasterev, Valentina Siracusa, Olga K. Karyagina, Sergey M. Lomakin, Tuyara V. Petrova, Levon Yu. Martirosyan, Anna B. Nikolskaia, Alexey L. Iordanskii

**Affiliations:** 1 Emanuel Institute of Biochemical Physics of Russian Academy of Sciences, 119334 Moscow, Russia; terj@rambler.ru (Y.V.T.); sergeykozlov1@gmail.com (S.S.K.); vpodmasterev@yandex.ru (V.V.P.); olgakar07@mail.ru (O.K.K.); lomakin@sky.chph.ras.ru (S.M.L.); levon-agro@mail.ru (L.Y.M.); anickolskaya@mail.ru (A.B.N.); 2 Department of Chemical Science (DSC), University of Catania, Viale A. Doria 6, 95125 Catania, Italy; 3 N.N. Semenov Federal Research Center for Chemical Physics Russian Academy of Sciences, 119991 Moscow, Russiaaljordan08@gmail.com (A.L.I.)

**Keywords:** biodegradation, soil, PLA/OTOA, antibacterial, packaging

## Abstract

Glycerol-(9,10-trioxolane) trioleate (OTOA) is a promising material that combines good plasticizing properties for PLA with profound antimicrobial activity, which makes it suitable for application in state-of-the-art biomedical and packaging materials with added functionality. In this study, the biodegradation kinetics of PLA + OTOA mixed films under soil conditions was assessed over 180 days. Structural and morphological changes that occurred on the surface and in the volume of the films during degradation were scrutinized using DSC, X-ray diffraction, IR, and UV spectroscopy. Morphological changes were assessed using optical and confocal microscopes. The different behavior of the PLA + OTOA blend films during decomposition in soil is explained by their structure and the rate of release of antibacterial OTOA from the PLA matrix. The decomposition rate constants were determined for all films, where k_d_ for PLA samples is 28 µm·year^−1^, for samples containing 10% and 30% OTOA k_d_ is 2 µm·year^−1^, and for PLA + 50% OTOA samples k_d_ = 34 µm·year^−1^. This is explained by changes in the structure and degree of crystallinity of materials during the process of aging in the soil. These results clarify the biodegradation processes of biomaterials containing antibacterial agents in their structure.

## 1. Introduction

A growing problem associated with the waste accumulation of difficult-to-decompose packaging polymer materials, constituting the bulk of daily household waste, has emerged in the last decade. The replacement of polymer materials with biodegradable polymers in packaging seems to be the optimal solution in order to reduce household waste [1,2]. In addition, much attention has recently been paid to the development of various packaging materials with additional functionality, such as antibacterial and antioxidant properties, designed to improve food safety, quality, shelf life, etc. [3]. In particular, the use of antibacterial packaging materials opens up new opportunities for longer food preservation without spoilage, while maintaining the quality and safety of food products [4,5].

The packaging must be strong and flexible and provide a barrier to gases, aromas and moisture, maintaining the optimal moisture balance necessary for the preservation of the product and its protection against various environmental influences. The antibacterial agents introduced into the polymer packaging material should additionally provide inhibition for the growth and spread of microorganisms that cause food spoilage [6,7]. Various antibacterial agents, such as chitosan [8], nanomaterials (e.g., silver nanoparticles) or natural compounds (e.g., plant extracts, essential oils, or polyphenols like resveratrol) were successfully introduced in the polymer packaging materials and showed a pronounced effect on slowing down the spoilage of food products [9,10,11,12]. Despite the successful examples of the development of the antibacterial food packaging based on biodegradable polymers (PLA, PHB, etc.), the problem of decomposition and biodegradation of such packaging in soil has arisen, since the antibacterial additives could, on the contrary, slow down its decomposition [9,13]. The studies of the degradation processes of biodegradable polymer materials can be carried out in various environments, such as soil, compost, water, and solid municipal waste [14,15]. The degradation mechanisms and the rate of polymer degradation could significantly vary, as they depend on many factors such as the type of the environment, soil composition, temperature, content of organic residues, and microorganisms [16,17,18].

Among biodegradable polymers used for packaging purposes, PLA stands out for its mechanical properties, non-toxicity, and biocompatibility [19]. PLA is also superior compared to synthetic polymer materials in terms of biodegradation rate, which could vary from months to several years depending on the degradation conditions (exposure in compost or soil, degradation temperature, and the amount of precipitates) [20]. Films made of pure PLA are fragile and require modifications in order to change its physicochemical properties and provide improved performance. For example, plasticization of PLA could improve elasticity and mechanical properties of PLA films. It should be noted that any modification of the reference PLA affects its physical, chemical, and morphological properties, and, therefore, its susceptibility to degradation [21,22].

Our previous studies showed that the addition of strong antibacterial agent, OTOA, has a positive effect on the physicochemical and mechanical properties of PLA films and non-woven materials due to the plasticizing effect of OTOA, which results in improved elasticity of PLA films, while maintaining their mechanical strength [23,24]. The study of the morphological, physicochemical, mechanical, and thermal properties of PLA films after adding different concentrations of OTOA revealed the optimal concentrations for various packaging and medical applications. Thus, PLA films containing 10% and 30% OTOA showed optimal mechanical and elastic properties, combining high elasticity and good tensile strength. OTOA plasticizes and increases the segmental mobility of PLA macromolecules, amorphizing the material and reducing its crystallinity, which contributes to changes in the thermodynamic and mechanical properties of PLA films. Since OTOA has pronounced antimicrobial activity, composite PLA + OTOA films have been studied in relation to antibacterial activity against various bacterial strains [23,25]. Additionally, the kinetics of OTOA release from PLA films was studied and the rates of OTOA release from PLA + OTOA blend films in physiological solution were estimated. It has been shown that the profile of OTOA release has two distinct stages, with the initial stage related to the relaxation of the PLA matrix and the second stage with an extremely low rate of OTOA release approximated by zero-order kinetics, which is attributed to the slow degradation of the PLA polymer [25]. Observed zero-order kinetics provides stable OTOA release from the PLA matrix, which is beneficial for various applications of OTOA-loaded PLA film materials (wound dressings, antibacterial food packaging, etc.).

Decomposition of PLA occurs by hydrolysis of ester bonds under the action of water, and its rate is largely determined by the diffusion of water molecules into the polymer volume and release of the reaction products into the environment [20,26]. The addition of plasticizers could enhance this first stage of PLA disintegration due to an increase in the mobility of the polymer chains and faster water diffusion inside the PLA matrix [11]. The degree of crystallinity is another important factor affecting the rate of hydrolytic PLA decomposition. Since the access of water molecules to denser crystalline areas of PLA is impeded, degradation first occurs in amorphous areas, which leads to an increase in the degree of crystallinity [20]. The size of the PLA crystallites and its orientation do not show a significant effect on the degradation rate at this stage. The degree of PLA crystallinity decreases only when the crystalline regions of the polymer material start to degrade [12,27]. It is important to note that microbial factors also affect PLA decomposition in soil. As soon as the microbial cells adhere on the PLA surface, the biodegradation process is launched with subsequent production of enzymes (proteases, lipases, esterases, and cutinases of microbial origin), which break the ester bonds in the PLA chain, forming PLA oligomers of various lengths [28,29]. Peroxide and hydroxyl radicals formed in the process of ester bond breaking could also participate in the process of PLA decomposition [30], while lactic acid formed during PLA hydrolysis could inhibit the growth and development of particular fungi and bacteria in the soil [31,32]. The rate of PLA decomposition could be additionally accelerated by adding excess microorganisms to the soil environment or by pretreating the PLA material before decomposition using thermal, photo-oxidative or chemical methods [33].

The production and application of PLA-based antibacterial packaging involve its further utilization in soil or compost environments. However, the improvements in the physicochemical, mechanical, and functional properties of PLA provided by particular antibacterial additives could reduce PLA biodegradability and the possibility of polymer waste utilization, since the antibacterial agent could inhibit the effect of microorganisms on the rate of PLA biodegradation [34,35,36]. Therefore, taking into account the constantly increasing share of PLA in the food waste stream [20], it is of great importance to relate the complex processes of PLA chemical and biodegradation in the soil environment to the physicochemical and morphological changes in the PLA matrix loaded with the various antibacterial additives. In order to provide comparative data for various PLA-additive combinations, it is necessary to study PLA degradation in laboratory conditions in standardized soil [37,38,39].

In this work, we studied the physicochemical changes and degradation processes for blended PLA films with different loadings of the OTOA antibacterial agent (10, 30, 50%) in comparison with the reference PLA film during the exposure in the soil for up to 180 days. The specific aspect of this work is based on the peculiar characteristic of OTOA, which simultaneously possesses plasticizing and antimicrobial properties. The interplay between these two features could greatly influence PLA degradation in solid bacterially containing medium and was addressed in this work. Noticeable structural and physicochemical changes occurred in the PLA films during exposure to the soil were detected by IR spectroscopy, DSC, and XRD. Morphological changes were studied using optical and confocal microscopy, while the turbidity of the PLA films was estimated by UV-Vis spectroscopy. The residual antibacterial activity of the PLA films after 180 days of exposure in the soil was also estimated. The results of this study could help to identify the relationships between the physicochemical properties of the PLA matrix, PLA-OTOA interactions, and the OTOA release process with the features of the PLA + OTOA films decomposition in the soil environment. The latter could provide important information on how the antibacterial additives affect the utilization properties of PLA materials, which is highly important for their extensive applications in biomedicine and food packaging.

## 2. Materials and Methods

### 2.1. Materials

NatureWorks Ingeo 3801X Injection Grade PLA (SONGHAN Plastics Technology Co., Ltd., Shanghai, China) with the average molecular weight of 1.9 × 10^5^ g/mol was used to obtain PLA and PLA + OTOA films. The polydispersity index (PDI) for PLA used in this study was about 1.8, which was determined, as described previously [40]. Glycero-(9,10-trioxolane)-trioleate (OTOA) was obtained from Medozon (Moscow, Russia), with a previously described chemical structure [40]. To prepare PLA solutions, dry purified chloroform (≥99.5%, Sigma-Aldrich Inc., St. Louis, MO, USA) was used. All reagents were used as received.

### 2.2. Preparation of PLA Films

All PLA films used in this work were prepared by solvent evaporation method from chloroform solutions, as described previously [24]. PLA (2 g) was dissolved in 50 mL of chloroform. Then, a certain amount of OTOA (10, 30 and 50 wt.% relative to PLA) was added to the PLA solution in chloroform and stirred for 12 h with a magnetic stirrer. After mixing, the obtained solutions were poured onto glass plates, and the films were dried to constant weight at ambient temperature. Pristine PLA film without additive was used as a control sample. The thickness of the films with different OTOA concentrations did not change significantly and had average values of 125 ± 50 μm.

### 2.3. Soil Experiments

**Figure 1:**
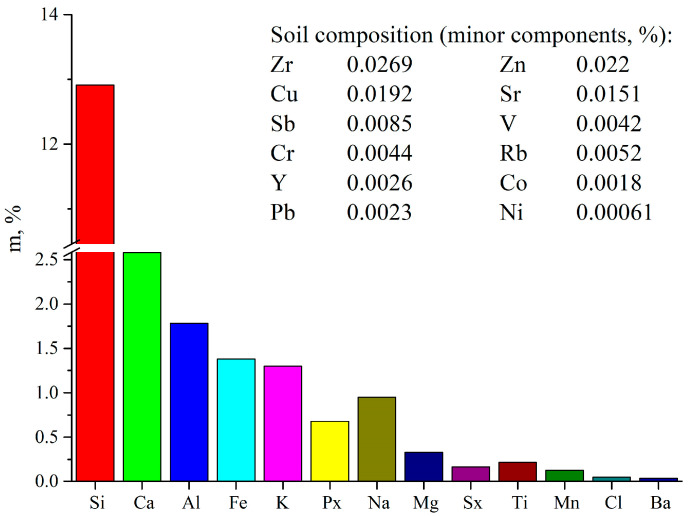
The soil composition before the biodegradation process.

Before the experiments, the soil properties and composition were characterized by pH and microelement composition. Soil samples for analysis were selected in accordance with ISO 13196 [41] and the chemical composition of soil was quantitatively determined according to ISO 11464 [42]. Soil pH was measured in accordance with ISO 21268-4:2019(en) [43] and appeared to be neutral. The soil composition was determined before the onset of the biodegradation process (Figure 1). The analysis results show that the soil was predominantly silicon rich (sandy component), and the content of mesoelements (Ca, Al, Fe) was also high. The content of microelements (Mn, Cu, Zn) was average, and all other elements are present in insignificant quantities.

During the study, the soil moisture was maintained in the range of 58–65%, providing optimal water-to-air ratio. Soil aeration was performed 2–3 times per month by puncturing the soil cover to a depth of 5 cm. Microorganism-rich soil with a microbial load of 9–10 × 10^9^ per gram was used for the soil experiments.

PLA film samples of 3 cm × 3 cm in size were buried in standardized soil at a depth of about 5 cm in groups (3 pieces of each type per 4 film types) for each single time point of aging. The distance between the samples was approximately 10 cm. After 40, 60, 120, and 180 days of aging in the soil, film samples were removed from the soil, washed with distilled water, dried at 40 °C to constant weight, and transferred for further physicochemical analysis. Three replicates were used for each film type at a single time point of aging.

### 2.4. Morphology and Opacity of PLA Films

The surface morphology of PLA and PLA + OTOA films before and after exposure in soil was studied using an Olympus CX21 optical microscope (Olympus Corp., Tokyo, Japan) and further processed using MICAM 3.02 software. PLA films morphology was also investigated using laser confocal microscope Olympus OLS5000 (Olympus Corp., Tokyo, Japan) and the 3D image of the sample surface, as well as the geometric dimensions of the structural elements on the film surface, were obtained. Images were acquired in the optical mode, and the relief map and surface image were obtained using laser scanning at 100× magnification.

The opacity of PLA films was probed using Shimadzu UV-3600 spectrophotometer (Shimadzu Europa GmbH, Duisburg, Germany) and was calculated using the following equation:

Opacity (mm^−1^) = *A_600_*/*X* (1)

where *A_600_* is the absorbance of the film at 600 nm and *X* is the thickness of film sample (mm) [44]. The thickness of the PLA films was measured using the digital micrometer and presented as the average value ± SD for at least 10 measurements at different sites on the film.

### 2.5. FTIR Spectroscopy Measurements

FTIR spectra of PLA films before and after exposure in soil were obtained, as described previously [24,25]. Bruker Tensor 27 IR Fourier spectrometer (Bruker Corporation, Billerica, MA, USA) was used, equipped with a PIKE MIRacle ATR accessory with a Teflon cell and germanium crystal (PIKE Technologies, Madison, WI, USA), which allows the measurements of solid samples. PLA film samples were tightly pressed to the surface of the Ge crystal in order to ensure good optical contact. FTIR spectra were recorded in the 400–4000 cm^−1^ range with 4 cm^−1^ resolution using an average of 16 consecutive scans.

### 2.6. X-Ray Diffraction Analysis

PLA and PLA + OTOA films before and after exposure in soil were studied by XRD using a DRON-3M X-ray diffractometer (Burevestnik, St. Petersburg, Russia) in the 2θ range of 10–40°, as described previously [25]. Relative crystallinity of the films was estimated as

*χ* = *I_C_*/(*I_C_* + *I_A_*) (2)

where *I_A_* and *I_C_* are the integral intensities corresponding to the respective amorphous and crystalline phases [45]. The relative error for *χ* (XRD) determination does not exceed 5%.

### 2.7. Differential Scanning Calorimetry

Thermal properties of the PLA and PLA + OTOA films were probed, as described previously [23,24], with the Netzsch DSC 204 F1 Phoenix differential scanning calorimeter (Netzsch, Selb, Germany) in the temperature range of 20–200 °C, at 10 °C/min heating rate and in the inert Ar atmosphere. Indium, tin, and lead were used for instrument calibration. Due to the high exothermic effect associated with the decomposition of OTOA and subsequent volatilization of its decomposition products, only one heating step was performed in DSC experiments in order to characterize the thermal properties of originally obtained PLA/PLA + OTOA films without “erasing their thermal history”. The degree of crystallinity (*χ*) for the studied films was calculated from DSC data according to the following equation:

(3) $$\chi =\frac{\Delta H_{m}-\Delta H_{cc}}{\Delta H_{m}^{100}\times \left(1-\beta \right)}\times 100\%$$

where ∆*H_m_* is the experimental melting enthalpy; Δ*H_cc_* is the enthalpy of cold crystallization; $\Delta H_{m}^{100}$ is the theoretical melting enthalpy of the 100% crystalline PLA (93.6 J/g); and *β* is the mass fraction of OTOA additive in the film. Deconvolution of complex DSC peaks obtained for the PLA + OTOA films was performed using NETZSCH Peak Separation 2006.01 program employing the Fraser–Suzuki algorithm for asymmetric DSC curves, as was described previously [23,24].

### 2.8. Weight Loss

PLA film samples with the dimensions of 3 × 3 cm were buried in soil (moisture content of 30% by weight) at a depth of 5 cm and were retrieved after 40, 60, 120, and 180 days. The obtained samples were washed with distilled water, dried to constant weight under vacuum at 40 °C and were stored in the desiccator. Equation (4) was used to calculate the percentage of weight loss [46] as follows:

(4) $$Weight\;loss\;(\%)=\frac{W_{i}\;-\;W_{t}}{W_{i}}\times 100\%$$

where *W_i_* is the initial weight of the film and *W_t_* is the dry weight of the film after exposure in soil for 40, 60, 120, and 180 days [47].

### 2.9. Measurements of Residual Antibacterial Activity

The antibacterial activity of PLA and PLA + OTOA film samples against *E. coli* before and after incubation in soil was measured by the Murray paper disk method [48,49]. *Escherichia coli* strain from the Korean Cell Line Bank was used in the experiments. Briefly, 100 µL of the culture medium of the strain was evenly spread on TSA (tryptic soy agar). After adding bacteria to the TSA medium, the culture was enriched for 18–24 h at 35–37 °C. The inoculum was prepared from an 18–20 h agar culture in meat peptone broth, bringing the turbidity to 0.5 McFarland standard. The resulting broth culture was diluted 10 times with a sterile isotonic NaCl solution, which corresponded to a final concentration of around 1 × 10^7^ CFU/mL. Inoculum was applied to Petri dishes with a dense nutrient medium using sterile cotton swabs. Thereafter, disks (6.0 ± 0.1 mm in diameter) were cut from PLA and PLA + OTOA films and applied to the seeded surface using sterile tweezers. Afterwards, the dishes were incubated for about 20 h at 37 °C. At the end of incubation, the retention of the visible growth zone was calculated based on complete inhibition of visible growth [50]. The experiments were repeated in triplicate. The antifungal activity against *Trichoderma harzianum* fungi was measured similarly; the Sabouraud agar was used as a growth medium.

## 3. Results

### 3.1. Morphology

**Figure 2:**
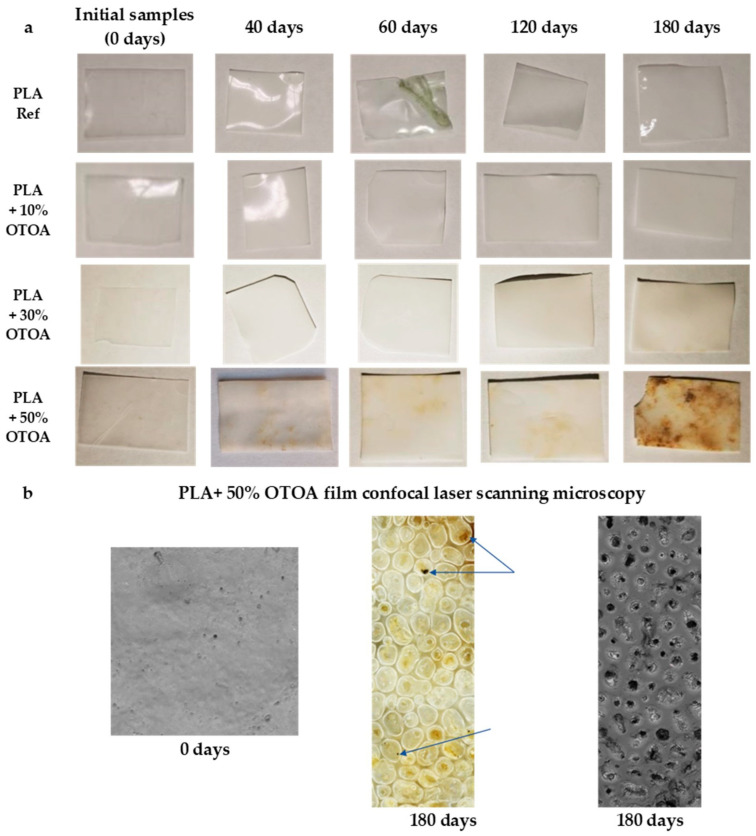
Images of PLA samples rendering visual changes occurring in the films after different times of exposure to soil (**a**); Images obtained with a confocal microscope at 100× magnification (**b**). Arrows indicate the traces of fungal spores at the film surface.

Morphological changes which happened in studied PLA films during incubation in soil were investigated using macro and microscopic observations. Macroscopic observations of the tested samples were useful for the initial assessment for the extent of the degradation process. The first stage of degradation is characterized mainly by changes in the color of the sample surface, since PLA films became less transparent and opaque (Figure 2). Additionally, color changes (appearance of the yellow tint) were visible for PLA films with 30% and 50% OTOA during exposure to soil. The sample with the greatest visual changes and the most obvious signs of degradation was the PLA + 50% OTOA sample after 180 days of storage in soil. Observed color change could be caused by both hydrolytic processes and the onset of the microbial action due to the presence of microorganisms on the surface of the PLA samples. Both factors could change the pH of the environment on the PLA surface, leading to changes in color and contributing to the degradation of the material.

**Figure 3:**
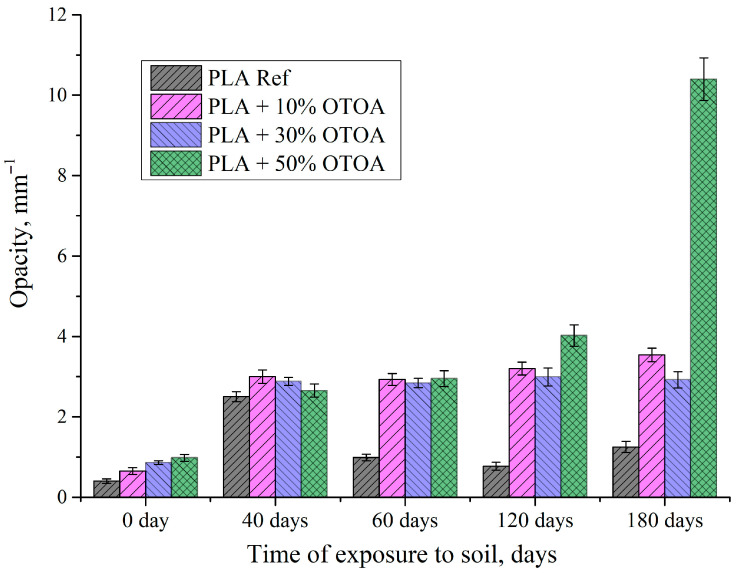
Changes in opacity of PLA films and PLA + OTOA films depending on the incubation time in soil.

One of the indicators of profound changes in the structure of PLA films occurring during degradation in soil is their opacity. Figure 3 shows the changes in the opacity of PLA and PLA + OTOA films during exposure to soil. Reference PLA film showed a sharp increase in the opacity after 40 days in soil and a further decrease at 60 days; thereafter, this parameter remained virtually unchanged over time. PLA films with 10% and 30% of OTOA also showed a sharp increase in the opacity values, which remained unchanged for up to 180 days, being in contrast to reference PLA. PLA + 50% OTOA film demonstrated a sharp increase in the opacity after 120 days in soil, which is associated with the onset of degradation due to hydrolysis processes and microbial activity. Observed changes in the opacity of the studied PLA films are in agreement with previously published data [51] and the initial increase in this parameter could be attributed to the increased crystallinity of PLA films. A decrease in the opacity observed for reference PLA film after 40 days in soil could be explained by the loss of crystallinity during prolonged exposure to soil.

Introduction of OTOA significantly changed the appearance and morphology of the PLA films, their microstructure, and surface properties (Figure 2 and Figure S1). Microscopic images of the tested samples before and after 40, 60, 120, and 180 days of degradation in soil are shown in Figure S1. Examination of the film microstructure after exposure to soil revealed a number of changes in film morphology, like the formation of pores (reference PLA film) and an increase in film roughness (PLA + 30% OTOA). PLA + 10% OTOA showed the most stable morphology during exposure to soil among the studied PLA film samples. For the PLA film with 50% OTOA, the formation of defects due to the release of OTOA and the onset of degradation processes, as well as the traces of fungal spores at the film surface, were observed (Figure 2).

The second stage of degradation of the PLA film materials, i.e., biofragmentation due to microorganism’s enzymatic attack, is characterized by the appearance of small/large holes on the surface of the polymer sample, which further leads to PLA disintegration. PLA + 50% OTOA film samples showed obvious signs of degradation and apparent fungal activity after 40 days in soil, including significant changes in film opacity, color, and roughness. PLA + 50% OTOA sample after 180 days of storage in soil showed the greatest morphological changes and the most discernible sings of biodegradation, revealing the disturbed morphology with visible defects and pores (Figure 2 and Figure S2). Figure S3 shows the distribution of pores and defects formed during degradation for the PLA + 50% OTOA sample after 180 days in soil. It could be seen that the bulk of the defects are about 100 nm in size. A photo obtained with a confocal microscope at 100× magnification also shows the presence of fungal spores deposited in the PLA + 50% OTOA matrix (Figure 2).

**Figure 4:**
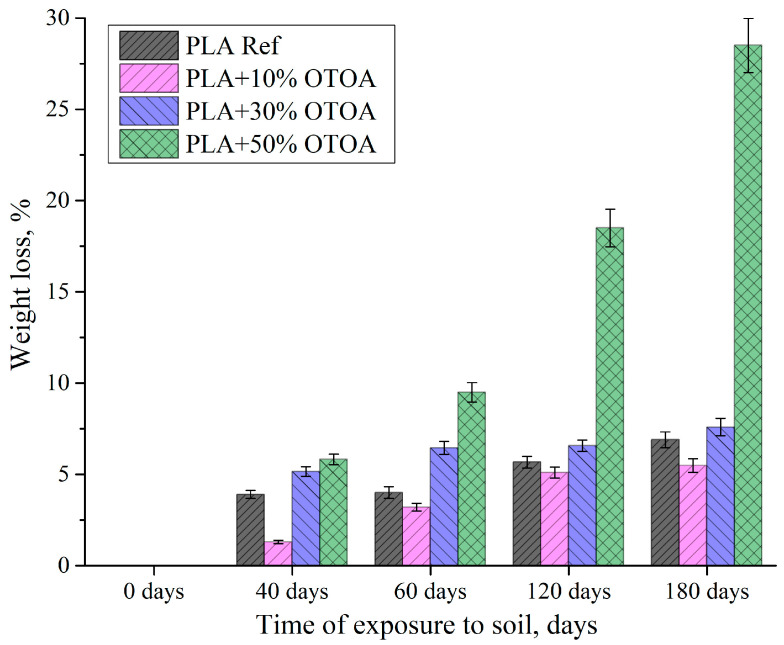
Weight loss percentage for studied PLA films vs. incubation time in soil.

One of the important indicators, which allows us to assess the presence of the degradation processes in polymer film samples, is the assessment of the weight loss during exposure to the soil. From Figure 4 it is evident that the most significant loss at the initial stage (40 days) was observed for the reference PLA film and PLA samples with 30% and 50% OTOA, while PLA + 10% OTOA film demonstrated the lowest weight loss. With further aging in the soil, the weight loss values reach a plateau by 180 days in soil for the reference PLA sample and PLA with 10% and 30% OTOA. In contrast, weight loss for the PLA + 50% OTOA film showed gradual increase, reaching the maximum value of as high as 28%. The observed pattern is apparently associated with the active release of OTOA from the PLA + 50% OTOA film and with the onset of hydrolytic destruction of the pristine polylactide, whose degradation subsequently proceeds at an approximately constant rate. It could be assumed that at the initial stage (up to 40 days), the release of ozonide from the PLA film with 10% OTOA does not make a significant contribution to the weight loss due to its initial small amount, but, at the same time, it could hinder the microbial activity during incubation in soil, which makes PLA + 10% OTOA film the most stable among the studied PLA samples.

**Figure 5:**
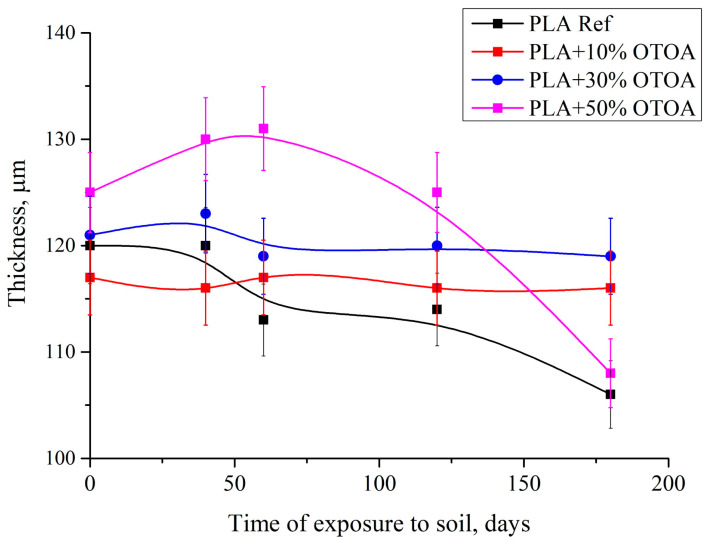
Changes in thickness of PLA films depending on the incubation time in soil.

From the graph presenting the changes in PLA film thickness vs. time of exposure to soil (Figure 5), it is evident that the main thickness changes were shown by the reference PLA film and PLA film containing 50% OTOA. This could be attributed to the fact that the reference PLA undergoes hydrolytic degradation, and the PLA + 50% OTOA sample loses antibacterial OTOA. On the contrary, PLA films containing 10% and 30% of OTOA were very stable in terms of film thickness.

According to Chamas et al. [52], the degradation rate of flat films is proportional to the surface area *S* (m^2^) and the rate constant *k* (kg∙s^−1^∙m^−2^) (Equation (5)).

(5) $$-\frac{dm}{dt}=k\cdot S$$

For thin films, the surface area (and rate) can be considered essentially constant for most of the degradation time. Generally, degradation occurs only on the flat surface of the polymer film, and the degradation rate is linearly proportional to the surface area (*S*). Dividing the rate constant (*k*) by the density of the polymer (*ρ*) yields the specific surface degradation rate (*k_d_*) with units of m∙s^−1^. The mass loss of polymer films is related to the decrease in their thickness by the following equation:

(6) $$-\;\frac{dm}{dt}\;=k_{d}\cdot \rho \cdot S$$

(7) $$\frac{m_{t}}{\rho \;\cdot \;S}=\frac{m_{0}}{\rho \;\cdot \;S}-k_{d}\cdot t$$

The expression *m*/(*ρ*∙*S*) in Equation (7) allows the average film thickness to be calculated from the mass loss. The *k_d_* values were calculated based on the difference in film thickness at the beginning and after 180 days of incubation in soil. For the pristine PLA sample *k_d_* was 28.0 µm∙year^−1^, for the PLA containing 10% and 30% OTOA *k_d_* was 2.0 µm∙year^−1^, and for the PLA + 50% OTOA sample *k_d_* was 34.0 µm·year^−1^.

Macroscopic observations of the studied PLA film samples after exposure to soil showed that the reference PLA sample demonstrated only minor changes in the opacity without loss of gloss, with some decrease in the film thickness during aging in soil. PLA samples with 10% and 30% OTOA behaved quite similarly in terms of opacity, with an increase in surface roughness. Additionally, PLA film containing 30% OTOA showed traces of fungal activity during aging in soil. PLA + 10% OTOA film appeared to be most stable among the studied PLA samples, while PLA + 50% OTOA film showed the highest weight loss during incubation in soil, accompanied with the biggest morphological changes and the most discernible sings of biodegradation. Although mechanical tests were not performed due to the difficulties in reliable determination of mechanical properties of the PLA film materials after storage in soil, an increase in film brittleness was observed with increased in time in soil, with the samples being fractured even with the slight force applied. This is especially evident for the PLA + 50% OTOA sample after 180 days in soil, where the edge is broken off (Figure 2). This result is similar to that obtained previously [53], where it was reported that the PLA films became brittle after only one month in soil. Observed significant changes in the morphology and microstructure of PLA films could be undoubtedly associated with substantial changes in the physicochemical properties of PLA films due to the processes of OTOA release, PLA hydrolysis, and biodegradation processes under the action of bacteria and fungi found in the soil.

### 3.2. FTIR-Spectroscopy

**Figure 6:**
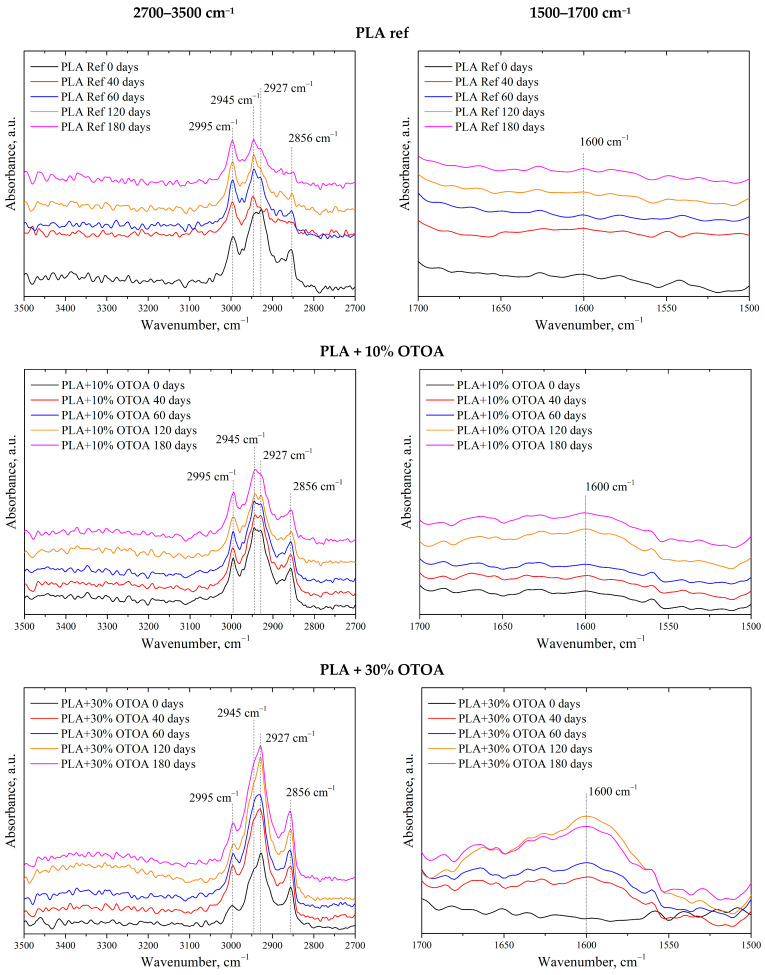

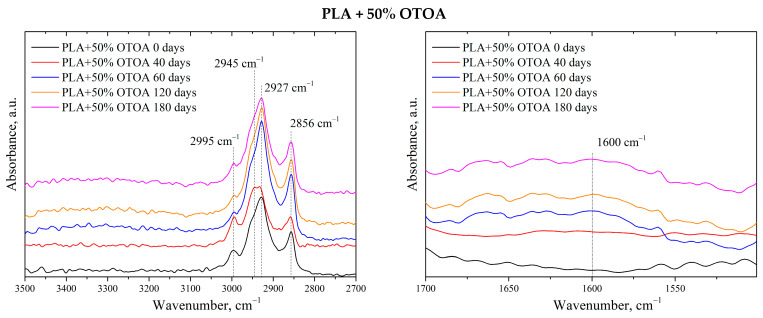
FTIR-spectra of reference PLA and PLA + OTOA films after different incubation times in soil: close-up view of FTIR spectra at 2700–3500 cm^−1^ interval (left column); close-up view of FTIR spectra in the 1500–1700 cm^−1^ interval (right column).

Full FTIR spectra for all PLA samples after 0, 40, 60, 120, and 180 days in the soil are shown in Figure S4. Figure 6 shows FTIR spectra of the reference PLA film and PLA + OTOA films after 0, 40, 60, 120, and 180 days in the soil in the wavenumber ranges of 2700–3500 and 1500–1700 cm^−1^. FTIR spectrum of the reference PLA film shows characteristic bands at 1455 and 1756 cm^−1^, which arise from the bending vibrations of –CH_3_ and the stretching vibrations of the C=O groups, respectively [54,55]. The absorption bands at 2944 cm^−1^ and 2995 cm^−1^ were attributed to the asymmetric stretching vibrations of the –CH group.

Two additional bands at 2927 cm^−1^ and 2856 cm^−1^ appeared in the spectra of PLA + OTOA films, which could be attributed to the stretching vibrations of –CH_2_ group [56,57]. Since PLA does not contain –CH_2_ groups in its chemical structure, while they are abundant in OTOA, the presence of these bands in the FTIR spectra proves the inclusion of OTOA in the supramolecular structure of PLA. As could be seen, the intensity of the absorption band at 2856 cm^−1^ correlates well with the OTOA content in PLA films. Comparison of the spectra before and after degradation in soil showed the formation of a new band at 1600 cm^−1^, which corresponds to vibrational band of carboxylate ions. This band is most pronounced in the spectra of PLA + OTOA films after 120 and 180 days in the soil. The appearance of carboxylate ions is attributed to the activity of microorganisms on the PLA surface that consume and break down lactic acid and its oligomers, leading to the formation of the carboxylates at the end of the PLA chains [58].

However, it is necessary to take into account that both hydrolytic processes and the process of OTOA release from the PLA matrix are also accompanied by partial destruction of the OTOA ozonide structure and the formation of acidic –COO groups. This is also evidenced by the appearance of additional bands around 1600 cm^−1^, which is clearly visible in the spectra in the 1500–1700 cm^−1^ range. Thus, the pristine PLA does not show a noticeable increase in the 1600 cm^−1^ region, while PLA films with 10%, 30%, and 50% OTOA showed a significant increase in the intensity of these peaks at different times of exposure to soil.

An increase in the intensity of the characteristic bands at 3160–3620 cm^−1^, related to the vibrations of -OH groups, was also observed for the studied PLA + OTOA samples [59]. The increase in the intensity of these bands correlates with the increase in the time of incubation in the soil. For the PLA + 10% OTOA film, an increase in the intensity of the band at 2945 cm^−1^, attributed to the stretching of the C-H bond of the –CH_3_ group, was observed, indicating the formation of additional –CH_3_ groups during incubation. Based on the analysis of the FTIR spectra, it could be assumed that both the PLA matrix and the OTOA molecules undergo slow hydrolysis and slow biodegradation during incubation in the soil.

### 3.3. DSC Analysis

**Figure 7:**
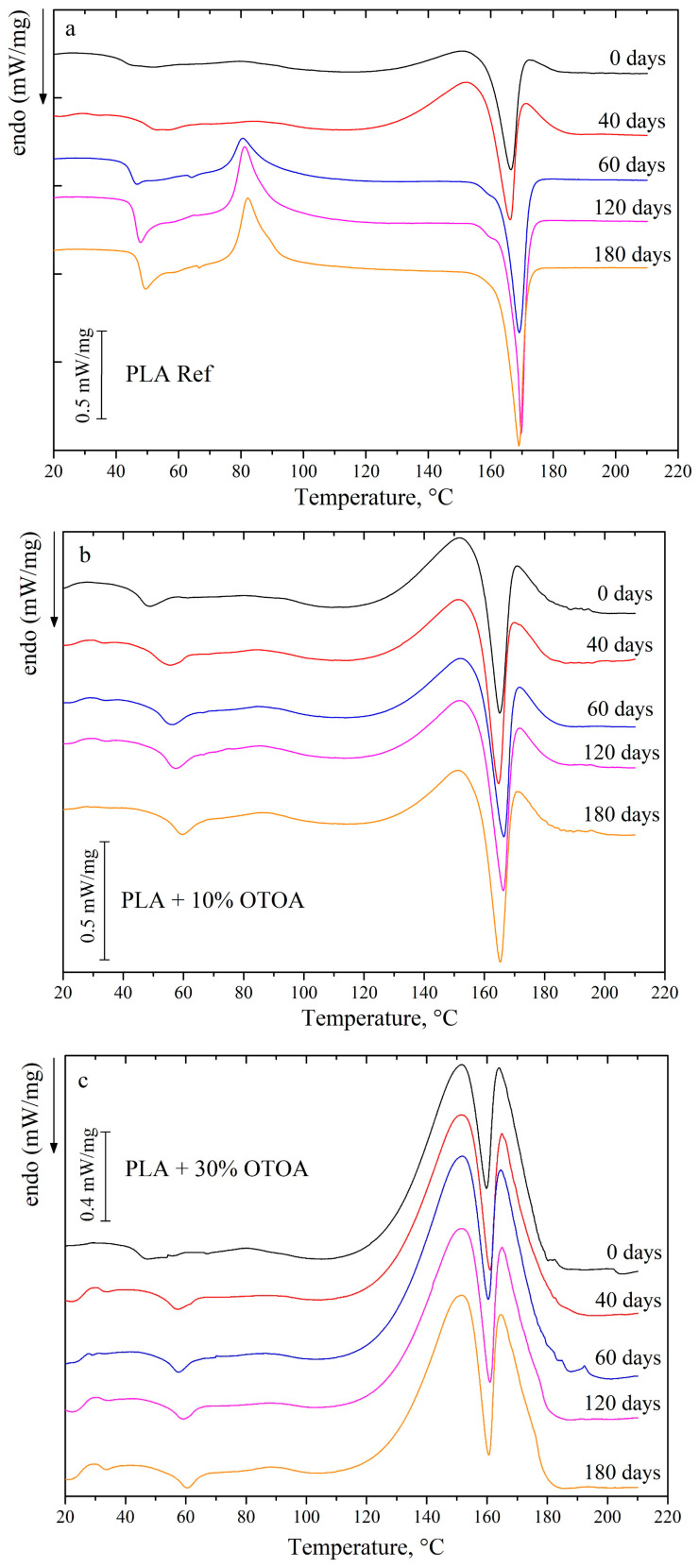

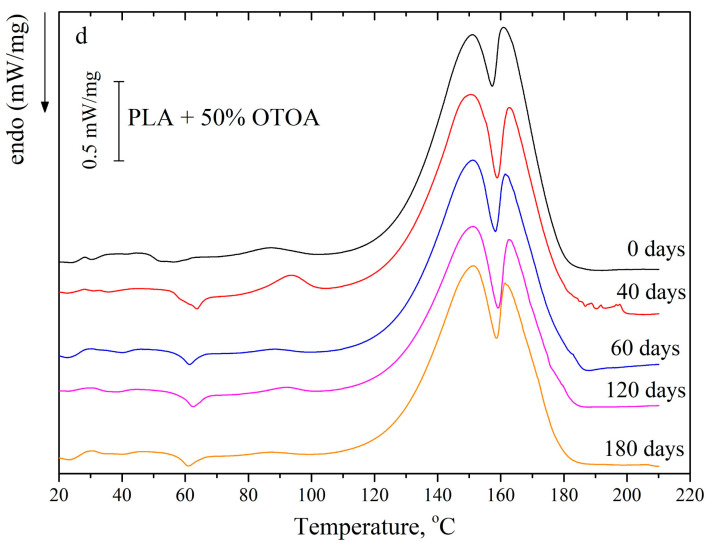
DSC thermograms of the reference PLA (**a**) and PLA + OTOA (**b**–**d**) films after different incubation times in soil.

Figure 7 shows DSC curves for PLA and PLA + OTOA films before and after degradation in soil for 40, 60, 120, and 180 days. The DSC thermograms for the reference PLA films before incubation in soil and at 40 days of incubation show one endothermic peak, which could be attributed to the melting of PLA crystallites. At incubation times higher than 40 days, PLA cold crystallization peak is observed, which indicates the amorphization of the reference PLA film during exposure to soil and formation of the disordered semi-crystalline PLA structure [60,61,62,63]. This structure could easily reorganize during heating at temperatures above the glass transition temperature with the formation of the additional PLA crystals, which is manifested in the increase in the cold crystallization enthalpy Δ*H_cc_* and the melting enthalpy Δ*H_m_* for the reference PLA film after incubation in the soil for more than 60 days. The degree of crystallinity in this case decreases from 22.9 to 11% (Table 1).

The thermodynamic characteristics for studied OTOA-added PLA films change depending on the time of incubation in soil. As was shown previously, the process of OTOA release is first observed for PLA + OTOA films during incubation, which also could contribute to the decrease in the amorphous part of the PLA films and therefore influence the degree of crystallinity. Since OTOA is a strong antibacterial agent, its release leads to the inhibition of the microbial contribution to the overall decomposition process during incubation in soil.

As could be seen from Figure 7 and Table 1, the thermodynamic characteristics for the PLA + 10% OTOA film (cold crystallization enthalpy Δ*H_cc_*, melting enthalpy Δ*H_m_*, degree of crystallinity) were stable during exposure to soil and only after 120 days of incubation the degree of crystallinity started to increase, which indicates on the degradation of the amorphous phase and/or partial OTOA release from the film. After 180 days in soil, no significant destruction of the PLA + 10% OTOA film was observed. Since OTOA acts as the plasticizer, it could increase the mobility of the PLA chains in the amorphous phase [23]. On the other hand, the hydrophobic nature of OTOA prevents increased water diffusion inside the amorphous PLA matrix, thus stabilizing the semi-crystalline PLA phase [11]. Thus, PLA + 10% OTOA film showed the slowest degradation rates of all the studied samples.

PLA films with 30% and 50% OTOA behave differently in the soil. The PLA + 30% OTOA film after 40 days in soil already shows an increase in the degree of crystallinity, which remains stable for up to 180 days. This could be attributed to the rapid release of the amorphous OTOA from the inter-spherulite space during the first 40 days of incubation, which leads to increased *χ* values. On the other hand, stability of the crystallinity values up to 180 days of incubation in soil confirms OTOA action on bacteria and fungi in the soil, which prevents further decomposition of PLA crystals at prolonged incubation times [64]. Additionally, a stabilization effect on the PLA semi-crystalline phase due to the hydrophobic nature of OTOA also takes place.

Thermodynamic characteristics for the PLA + 50% OTOA film were significantly changed during incubation in soil. Initially, PLA + 50% OTOA film showed a low degree of crystallinity, around 11%, and substantial cold crystallization enthalpy Δ*H_cc_* (4.9 J/g). After 40 days in soil, the cold crystallization enthalpy was increased almost two-fold, indicating on the amorphization of the PLA + 50% OTOA film during exposure to soil. This could be possibly due to the fast degradation of the amorphous parts of the PLA matrix, since addition of 50% OTOA leads to formation of the distorted semi-crystalline PLA structure. At 60 days in soil and thereafter, the degree of crystallinity for the PLA + 50% OTOA film increased almost two-fold, mostly due to the process of OTOA release from the PLA matrix. After 120 days in soil, the process of PLA crystals decomposition could be observed, which is manifested in the decrease in the degree crystallinity for the PLA + 50% OTOA film. This could be correlated with the PLA degradation due to the action of the fungi and bacteria in the soil [65].

### 3.4. XRD

**Figure 8:**
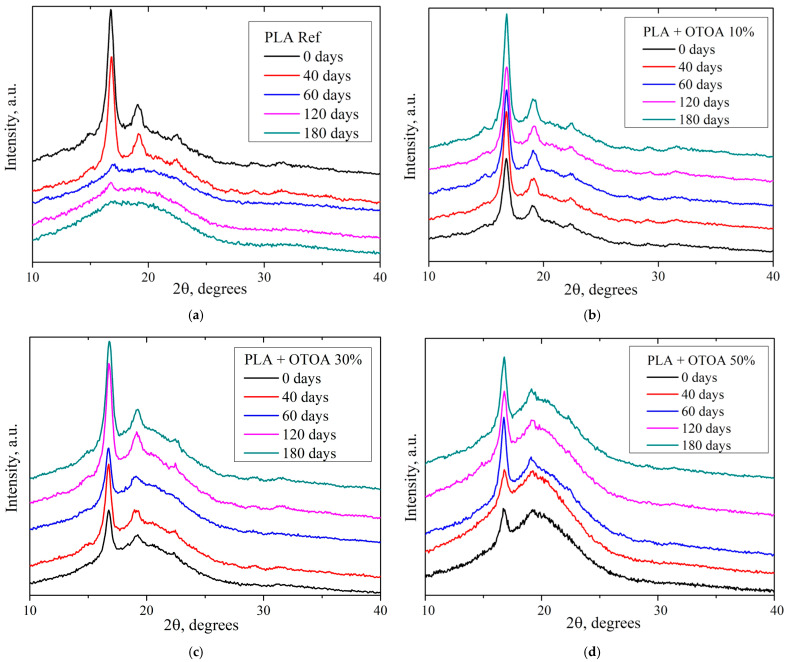
XRD patterns of reference PLA film (**a**), PLA + 10% OTOA (**b**), PLA + 30% OTOA (**c**) and PLA + 50% OTOA (**d**) films obtained before and during incubation in soil.

To analyze changes in the crystalline structure of polymer films as a result of their biodegradation in soil, the XRD method was used. Figure 8 shows the XRD diffractograms for the PLA film samples before and after incubation in soil. Pristine reference PLA film (w/o incubation) showed intense diffraction peaks at 16.8° and 19.1° 2θ, which correspond to the diffractions of (200)/(110) and (203) planes, and a smaller peak at 22.5° 2θ related to the (210) reflection [66]. The absence of the (213) reflection at 24.1° 2θ suggests that the primary crystalline phase for the pristine reference PLA film was the α’ phase [67]. The degree of crystallinity for the reference PLA film prior to incubation was 29.5% (Table 2), which is similar to the values observed previously [18,20]. XRD of the reference PLA (Figure 8a) showed a small decrease in the degree of crystallinity after 40 days of incubation, while a drastic decrease in the crystallinity of the PLA films was observed after 60 days of exposure (Table 2). By 180 days in soil the reference PLA film appeared completely amorphous, showing wide halo at 10–30° 2θ [66,67,68,69,70]. Observed amorphization for the reference PLA samples after 60 days of storage in soil is not correlated well with the degree of crystallinity obtained from DSC data. This could be accounted for the formation of the disturbed PLA semi-crystalline structure during decomposition of the reference PLA films in the soil, which is characterized by the absence of the long-range order (i.e., crystallinity observed by XRD), with the presence of small highly disturbed PLA crystals uniformly distributed inside the PLA film. These small crystals could give contribution in the overall DSC signal during melting and thus provide the discrepancy in the degree of crystallinity values obtained for the reference PLA films from XRD and DSC data. Formation of such a structure during incubation in soil for the reference PLA film could be accounted for the initial hydrolysis of amorphous phase and subsequent fragmentation and disintegration of the crystalline phase due to increased water diffusion.

Pristine PLA films with 10% and 30% OTOA showed intense diffraction peaks at 16.8° and 19.1° 2θ, while the intensity of the (210) reflection at 22.5° 2θ was small, which could be attributed to the formation of a more disturbed PLA α’ phase as a result of OTOA addition. This was even more pronounced for the pristine PLA + 50% OTOA film, which showed only 16.8° peak in the XRD pattern (Figure 8d).

PLA films with various amounts of added OTOA generally showed an increase in the degree of crystallinity during incubation in soil (Table 2). Obtained *χ* values were in good correlation with the crystallinity values obtained from DSC. Thus, the PLA + 10% OTOA films showed only slight increase in the crystallinity during incubation, which remained stable for up to 180 days, being in contrast with the reference PLA without OTOA. This could be attributed to the slow degradation of the amorphous phase due to the hydrophobic nature of OTOA, which acts as the plasticizer but prevents increased water diffusion inside the amorphous PLA matrix, leading to the stabilization if the semi-crystalline PLA phase.

PLA films with 30% and 50% OTOA content showed similar behavior during incubation in soil in terms of film crystallinity, showing the increase in the χ values after 40–60 days of storage in the soil, which remained stable for up to 180 days. This could be explained by the rapid release of OTOA from the amorphous parts of the films and the inter-spherulite space and further inhibition of crystalline phase decomposition due to the effect of OTOA on bacteria and fungi in the soil [64]. Generally, observed trends for the evolution of the χ values during incubation in soil as obtained from XRD and DSC, were similar for PLA films with 10%, 30% and 50% OTOA content.

### 3.5. Residual Antifungal and Antibacterial Activity

The residual antibacterial activity against *E. coli* was studied for pristine PLA films (0 days in soil) and on those that were incubated in soil for 180 days. The choice of *E. coli* was stipulated by its prevalence and possible antibiotic susceptibility profile with multidrug resistance [71,72]. Throughout the study, OTOA-added PLA film samples retained their antibacterial activity, despite the release of the OTOA from the films (Table 3). After 180 days of incubation in soil, the lysis zones for all samples were small, but there was no bacterial growth under the film sample due to the release of free OTOA from the PLA film. Observed results evidence that even the residual amounts of OTOA in the degraded PLA films could affect the activity of the bacteria.

The study of residual antifungal activity was carried out in relation to *Trichoderma harzianum* fungi [73]. Trichoderma fungi could influence the biodegradation of PLA films in soil, with some studies indicating that certain strains can accelerate the process [74,75,76].

**Figure 9:**
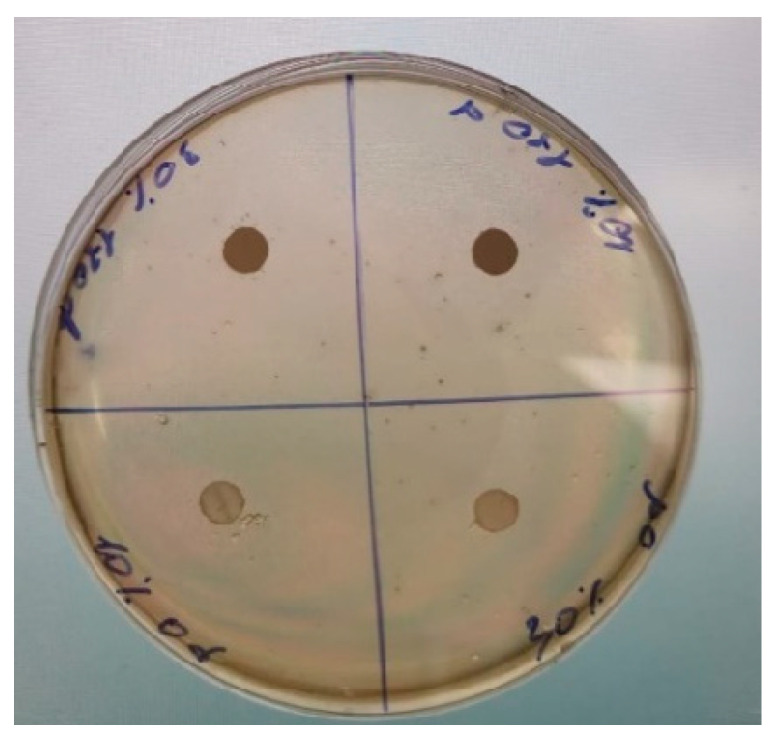
Comparison of the residual antifungal activity of the pristine PLA + 10% OTOA (lower left quarter) and PLA + 30% OTOA (lower right quarter) films and the same films after 120 days in soil (upper right quarter—PLA + 10% OTOA, upper left quarter—PLA + 30% OTOA).

The studied PLA + OTOA films (both before incubation and after 120 days in soil) did not show noticeable antifungal activity with visible inhibition zones (Figure 9). However, PLA film disks for the samples after incubation were significantly darker, as compared to the pristine ones. This could be rationalized as follows. *Trichoderma harzianum* produces significant levels of melatonin, which acts as an internal antioxidant and signaling molecule, with levels increasing dramatically under various stresses (like heavy metals, active oxygen species, etc.) [77]. Since the OTOA molecule contains ozonide groups and is a strong oxidizing agent, its interaction with the fungi could provide excess melatonin synthesis, leading to the dark disk color. For pristine PLA + OTOA films, most of the OTOA is not accessible for the *T. harzianum* fungi, since PLA + OTOA composite film retains its structure during test. Therefore, melatonin is produced at low levels, and the disk color is faint. PLA + OTOA films after exposure to soil possess degraded structure with more OTOA released from the PLA + OTOA composite film, thus the melatonin level is increased and the disk color is dark. Therefore, this test qualitatively indicates that PLA + OTOA film structure was degraded after soil exposure. Obviously, the release of OTOA from the films during incubation in soil affects the colonization of the films by fungal spores. Thus, it is possible to select the optimal concentration of OTOA content to accelerate or slow down PLA + OTOA film decomposition, taking into account the antibacterial and antifungal activity of the studied materials.

### 3.6. Conclusions

In this study, the degradation of the composite PLA films with different loadings of the OTOA antibacterial agent was comprehensively investigated during incubation in the soil for up to 180 days. The physicochemical properties of PLA and PLA + OTOA films were studied before and after incubation using DSC, XRD, and FTIR spectroscopy, and morphological changes were assessed using optical and confocal microscopy. Addition of OTOA, being simultaneously an antibacterial and plasticizing agent, into the PLA films significantly influenced the physicochemical properties of the PLA matrix, and, depending on the OTOA content, the peculiarities of the PLA + OTOA films decomposition in the soil environment.

The DSC and XRD showed that incubation in soil led to a complete amorphization of the reference PLA film and a formation of a more disturbed α’-phase in PLA + OTOA films due to partial hydrolysis of amorphous zones and/or the most unstable crystallites in the semi-crystalline PLA structure.

The observed increase in the crystallinity degree of the PLA + OTOA films during incubation in soil could be explained by the enhanced mobility of hydrolyzed PLA chains, leading to the formation of additional crystalline structures, the OTOA release from the PLA matrix, and the inherent bioactivity of the soil environment. Thus, PLA + 10% OTOA film showed only a slight increase in crystallinity during incubation, which remained stable for up to 180 days. This could be attributed to the slow degradation of the amorphous phase due to the hydrophobic nature of OTOA, which acts as the plasticizer but prevents increased water diffusion inside the amorphous PLA matrix, leading to the stabilization of the semi-crystalline PLA phase. Both PLA hydrolytic processes and the process of OTOA release from the PLA matrix are accompanied by the formation of acidic –COO groups, which is evidenced by the appearance of additional peaks at 1600 cm^−1^. Thus, the reference PLA did not show a noticeable peak in the 1600 cm^−1^ region during, whereas PLA + OTOA films showed a significant increase at the 1600 cm^−1^ band during soil exposure.

Residual antibacterial and antifungal activity for the PLA + OTOA films were assessed, as this may accelerate or retard the biodegradation of the studied materials. The PLA + OTOA films retained the antibacterial activity after 180 days of incubation, despite the release of the OTOA from the films. Although PLA + OTOA films did not show noticeable antifungal activity with visible inhibition zones, OTOA release from the PLA films after exposure in soil and its interaction with fungi leads to the excess melatonin synthesis as the response to the stress factor. Obtained results qualitatively evidence that PLA + OTOA film structure was degraded after soil exposure and were in good correlation with the FTIR spectroscopy data.

The different behavior of the PLA + OTOA films during decomposition in soil is explained by their structure and the rate of OTOA release from the PLA matrix. The decomposition rate constants k_d_ were determined as 28 µm·year^−1^ for the reference PLA film, 2 µm·year^−1^ for PLA films with 10% and 30% OTOA, while the k_d_ for the PLA + 50% OTOA sample was 34 µm·year^−1^. Obtained results could be explained by changes in the structure and degree of crystallinity of materials during the process of aging in the soil. These results clarify the biodegradation processes of biomaterials containing antibacterial agents in their structure and have the predictive merit for other modifiers of biodegradable polymers with the dual activity combining antibacterial and plasticizing properties.

## Figures and Tables

**Table 1:** Thermodynamic characteristics of studied PLA films and the degree of crystallinity (*χ*) of PLA films obtained from DSC data.

Reference PLA						
Time of Incubation in Soil, Days	T_g_ (°C)	T_cc_ (°C)	Δ*H_cc_* (J/g)	T_m_ (°C)	Δ*H_m_* (J/g)	*χ* (%)
0	45.3	79.5	3.8	166.4	25.3	23.0
40	53.5	83.9	2.9	166.2	25.7	24.4
60	46.7	80.6	14.9	169.1	32.8	19.1
120	47.7	81.2	26.0	169.8	35.1	9.7
180	49.4	82.2	24.9	169.0	35.7	11.6
**PLA + 10% OTOA**						
0	48.5	80.0	4.2	165.2	25.1	24.8
40	55.6	84.2	4.4	164.7	25.8	25.4
60	56.1	84.6	4.8	166.5	26.2	25.4
120	57.5	85.6	5.3	166.4	28.0	26.9
180	59.8	86.3	3.6	165.3	29.0	30.2
**PLA + 30% OTOA**						
0	46.0	80.1	5.5	159.8	16.5	16.7
40	57.0	85.7	1.4	161.0	19.2	27.2
60	57.6	86.2	2.9	160.4	18.2	23.3
120	59.0	88.1	1.3	161.0	18.9	26.8
180	60.6	88.0	2.1	161.0	19.7	26.9
**PLA + 50% OTOA**						
0	56.2	87.5	4.6	157.3	9.7	10.9
40	63.6	93.9	8.2	158.8	14.2	12.8
60	61.4	88.8	1.7	158.3	11.1	20.0
120	62.5	92.0	2.3	159.2	14.2	25.0
180	61.2	87.0	1.5	158.6	10.6	19.3

**Table 2:** Degree of crystallinity (*χ*) of PLA films with various amounts of added OTOA during incubation in soil obtained from XRD data.

Degradation Time, (Days)	0	40	60	120	180
PLA Film Sample	*χ* (%)				
PLA Ref	29.5	26.3	8.4	2.2	0
PLA + 10% OTOA	26.7	27.8	27.2	27.0	29.6
PLA + 30% OTOA	17.4	25.8	20.2	24.9	24.8
PLA + 50% OTOA	11.3	12.3	16.8	17.5	16.4

**Table 3:** Residual antibacterial activity of reference PLA film and PLA films with 10%, 30%, and 50% OTOA against *E. coli*.

Sample	Bacterial Strain	
	*E. coli*	
	Size of Clear Zone (mm)	
	0 days	180 days
Ref PLA	0.0 ± 0.0	0.0 ± 0.0
PLA + 10% OTOA	25.0 ± 0.2	6.1 ± 0.1
PLA + 30% OTOA	27.6 ± 0.3	6.1 ± 0.1
PLA + 50% OTOA	28.8 ± 0.2	6.2 ± 0.1
OTOA *	29.0 ± 0.1	

* Data obtained from ref. [18].

## Data Availability

The original contributions presented in this study are included in the article. Further inquiries can be directed to the corresponding authors.

## Supplementary Materials

The following supporting information can be downloaded at: https://www.mdpi.com/article/10.3390/polym18020216/s1, Figure S1: Optical microphotographs of the pristine PLA and PLA + 10, 30 and 50% OTOA films before and after different exposure times in soil; Figure S2: Confocal laser scanning microscopy of the pristine PLA film and PLA + 50% OTOA film before and after 180 days in soil. 3D image of the sample surface and measurement of the geometric dimensions for the structural elements on the surface. Images obtained in the optical mode and the relief map and surface image were obtained using laser scanning at 100× magnification; Figure S3: Distribution of pore sizes for PLA + 50% OTOA film after 180 days of incubation in soil; Figure S4. FTIR spectra of reference PLA and PLA + OTOA films after different incubation times in soil and FTIR spectra of PLA films after 180 days in soil at 2700–3700 cm^−1^ and 1500–1700 cm^−1^ intervals.
