# Supplementary material for: Evaluation of the Biodegradability Potential of Antibacterial Poly(lactic acid)/Glycero-(9,10-trioxolane)-trialeate Films in Soil
Source: Polymers (Basel). 2026 Jan 13;18(2):216. doi: 10.3390/polym18020216 (PMC12845568; doi:10.3390/polym18020216)

Supplementary Materials:

Morphology

Figure S1. Optical microphotographs of the pristine PLA and PLA + 10, 30 and 50% OTOA films before and after different exposure times in soil.

	0 days	40 days	60 days	120 days	180 days
Ref PLA					
PLA + 10% OTOA					
PLA + 30% OTOA					
PLA + 50% OTOA					

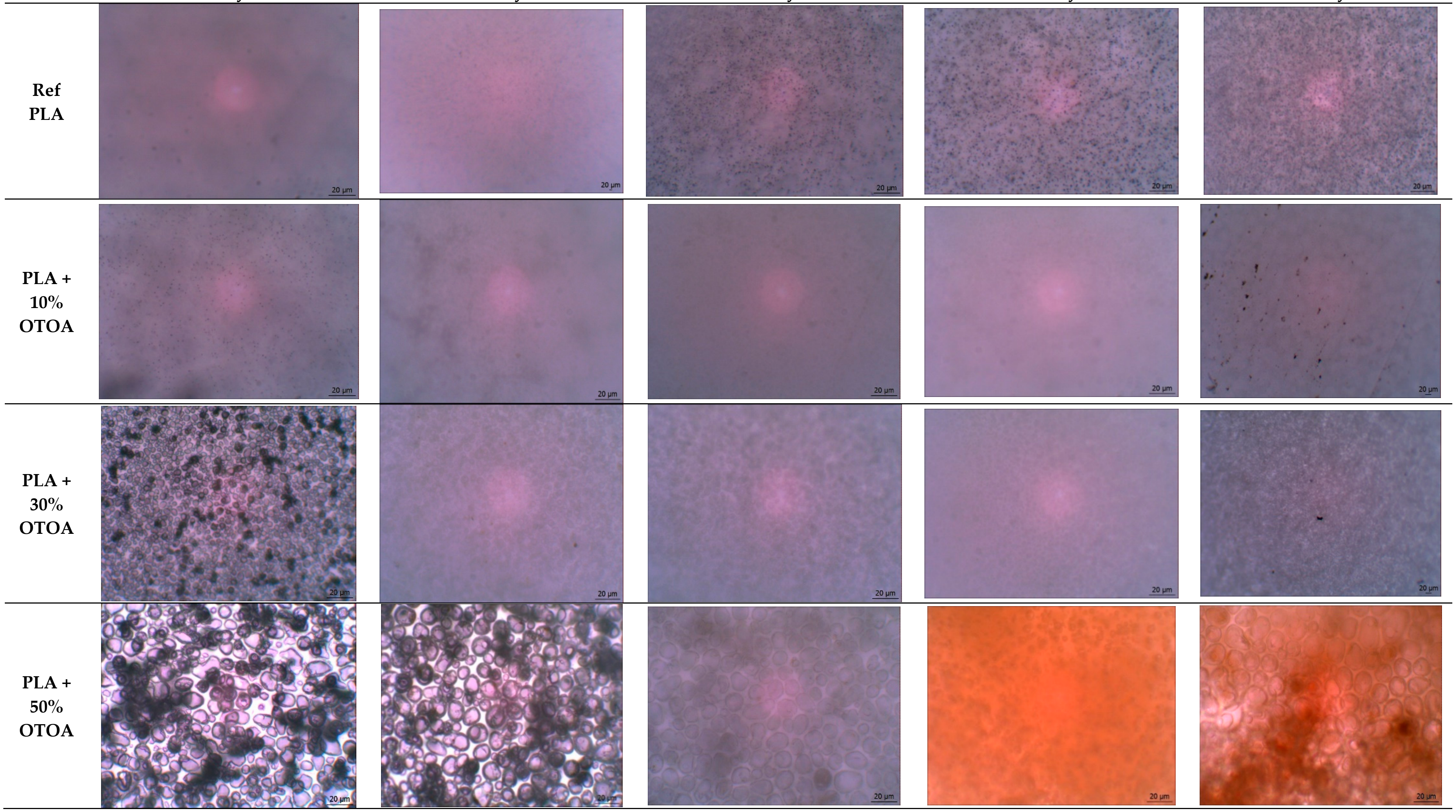

Figure S2. Confocal laser scanning microscopy of the pristine PLA film and PLA + 50% OTOA film before and after 180 days in soil. 3D image of the sample surface and measurement of the geometric dimensions for the structural elements on the surface. Images obtained in the optical mode and the relief map and surface image were obtained using laser scanning at 100x magnification.

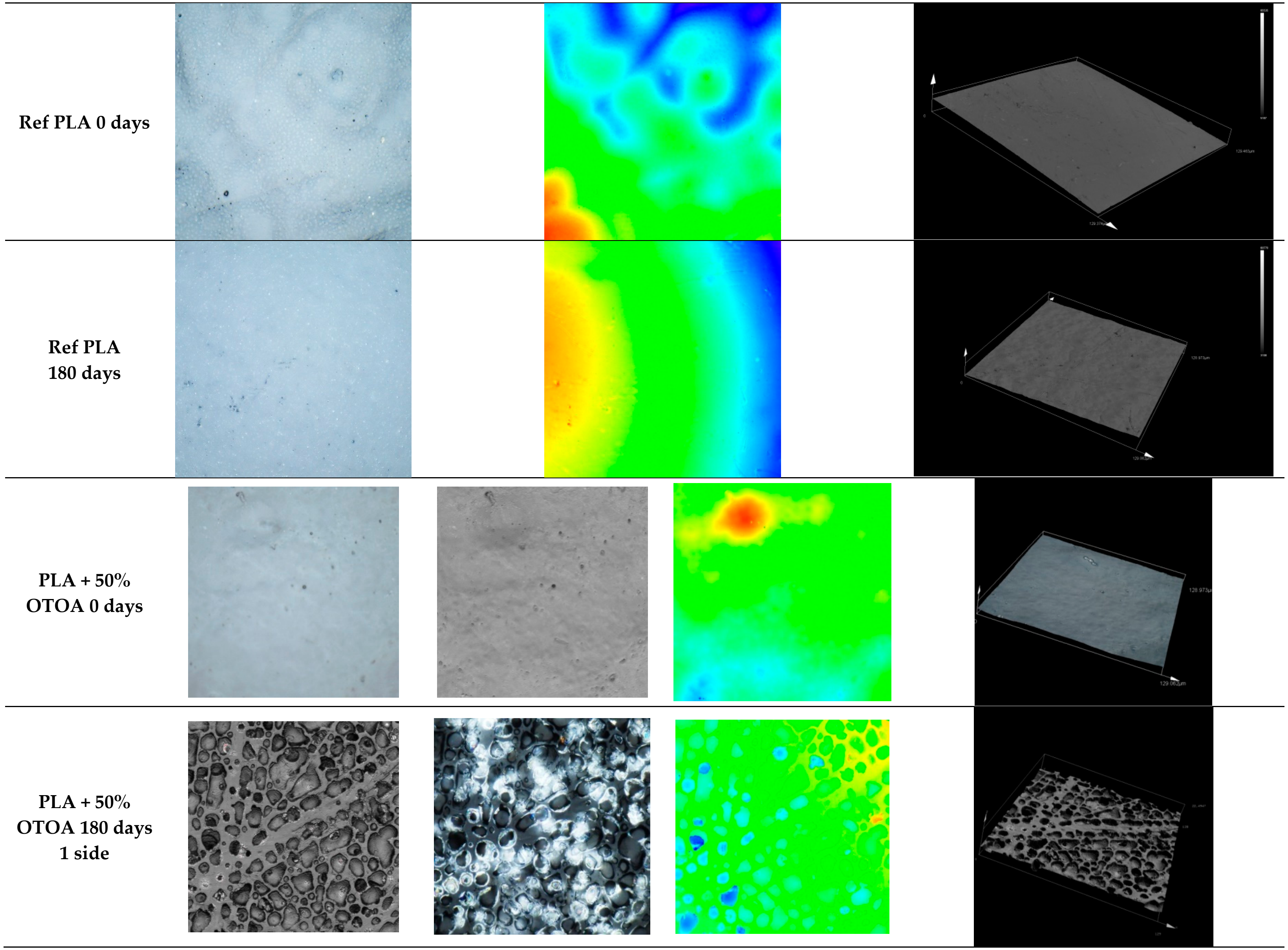

**PLA + 50% OTOA 180 days 2 side**	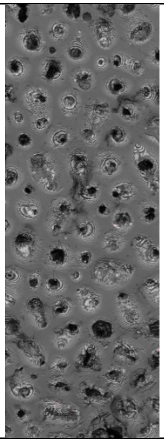	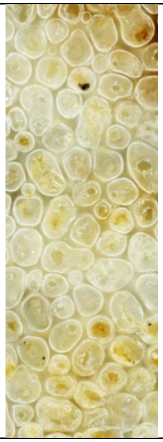	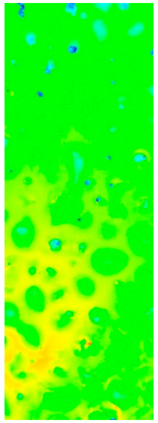	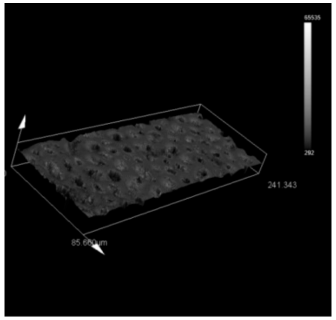

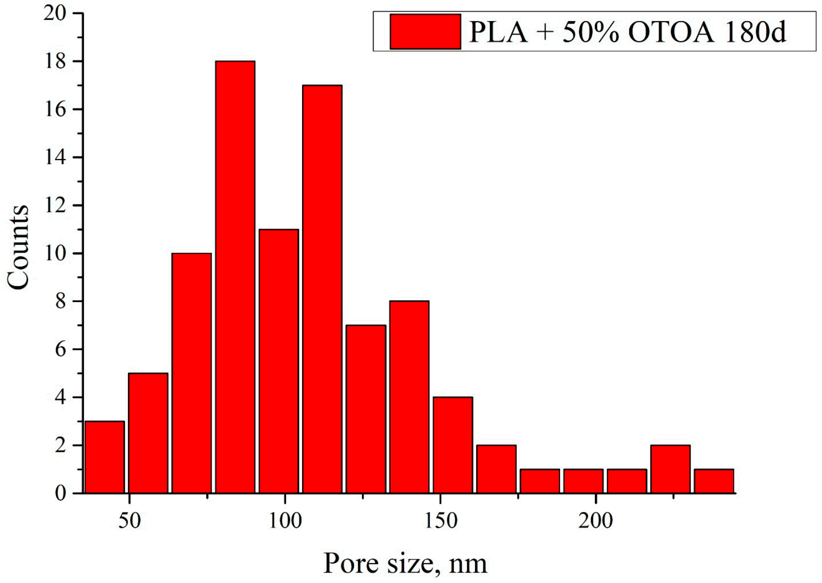

Figure S3. Distribution of pore sizes for PLA + 50% OTOA film after 180 days of incubation in soil.

Figure S4. FTIR spectra of reference PLA and PLA + OTOA films after different incubation times in soil and FTIR spectra of PLA films after 180 days in soil at 2700 – 3700 cm^{-1} and 1500 – 1700 cm^{-1} intervals.

<table>
<tr><th>PLA ref</th><th>PLA + 10% OTOA</th></tr>
<tr><td>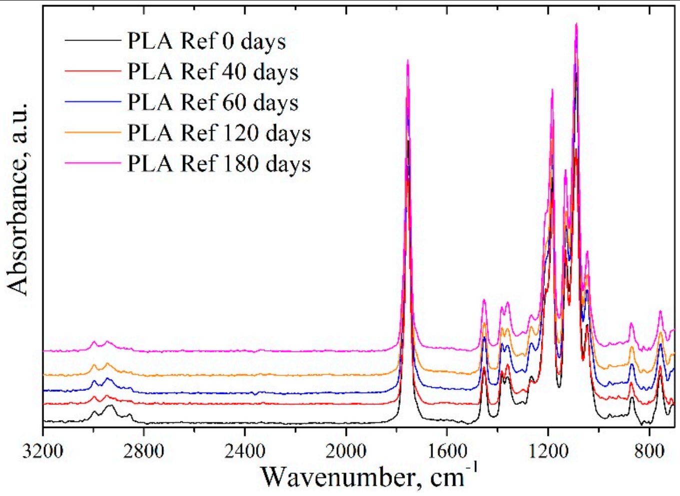
</td><td>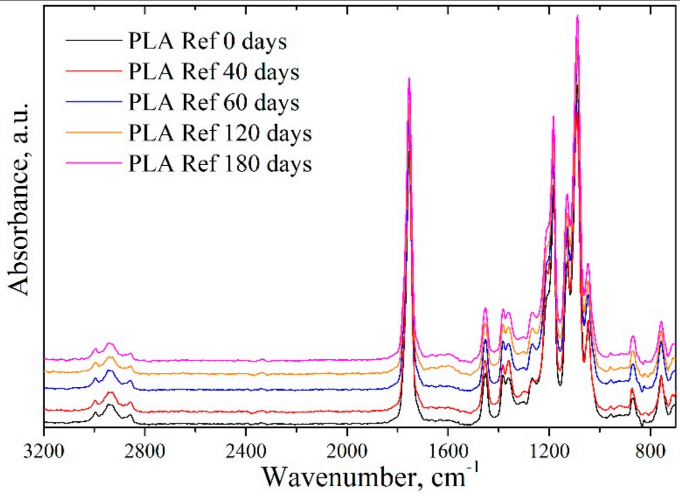
</td></tr>
<tr><th>PLA + 30% OTOA</th><th>PLA + 50% OTOA</th></tr>
<tr><td>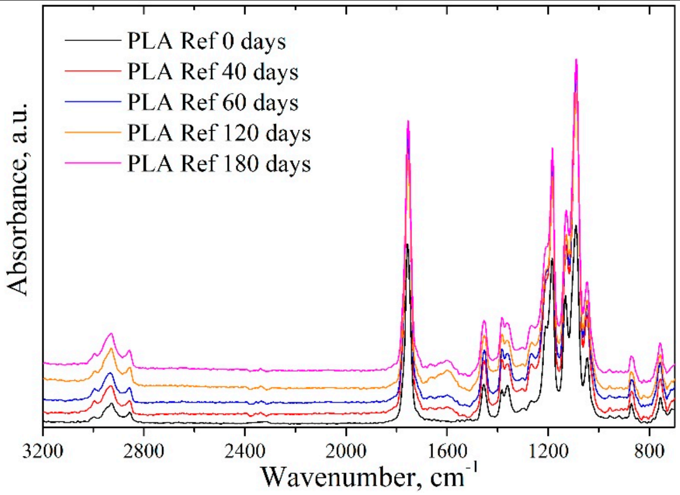
</td><td>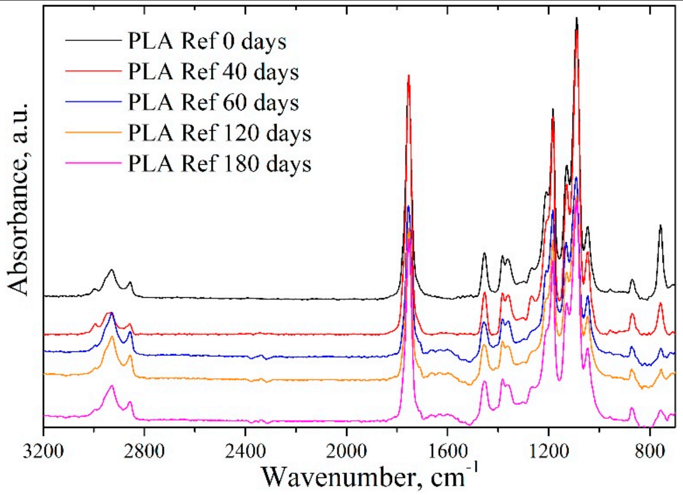
</td></tr>
</table>

PLA + OTOA 180 days

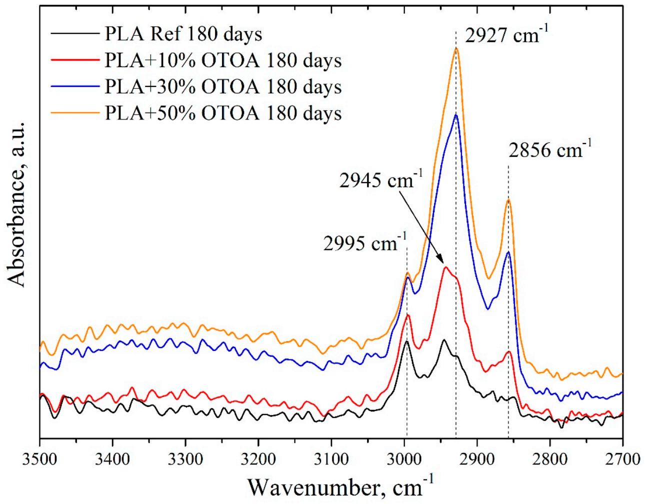

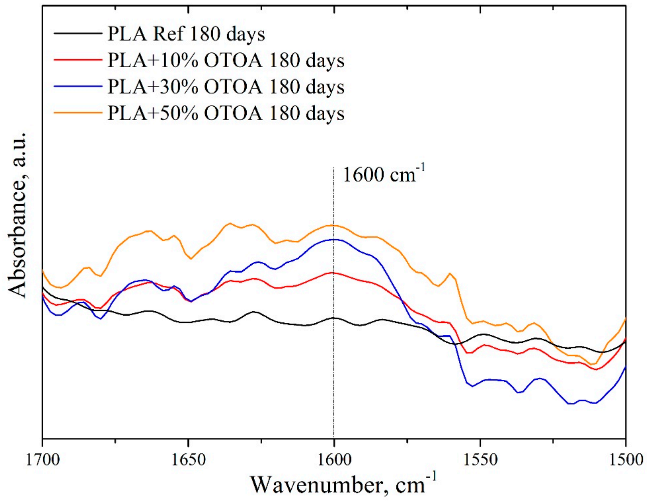

Supplement: Supplementary file 1 [file polymers-18-00216-s001.zip › polymers-4045031-supplementary.pdf]
